# Renewable Sources of Plant Biostimulation: Microalgae as a Sustainable Means to Improve Crop Performance

**DOI:** 10.3389/fpls.2018.01782

**Published:** 2018-12-07

**Authors:** Pasquale Chiaiese, Giandomenico Corrado, Giuseppe Colla, Marios C. Kyriacou, Youssef Rouphael

**Affiliations:** ^1^Department of Agricultural Sciences, University of Naples Federico II, Portici, Italy; ^2^Department of Agriculture and Forest Sciences, University of Tuscia, Viterbo, Italy; ^3^Department of Vegetable Crops, Agricultural Research Institute, Nicosia, Cyprus

**Keywords:** active molecules, plant-microbiome, modern agriculture, plant response, algae extracts, synergetic properties

## Abstract

Plant biostimulants (PBs) attract interest in modern agriculture as a tool to enhance crop performance, resilience to environmental stress, and nutrient use efficiency. PBs encompass diverse organic and inorganic substances (humic acids and protein hydrolysates) as well as prokaryotes (e.g., plant growth promoting bacteria) and eukaryotes such as mycorrhiza and macroalgae (seaweed). Microalgae, which comprise eukaryotic and prokaryotic cyanobacteria (blue-green algae), are attracting growing interest from scientists, extension specialists, private industry and plant growers because of their versatile nature: simple unicellular structure, high photosynthetic efficiency, ability for heterotrophic growth, adaptability to domestic and industrial wastewater, amenability to metabolic engineering, and possibility to yield valuable co-products. On the other hand, large-scale biomass production and harvesting still represent a bottleneck for some applications. Although it is long known that microalgae produce several complex macromolecules that are active on higher plants, their targeted applications in crop science is still in its infancy. This paper presents an overview of the main extraction methods from microalgae, their bioactive compounds, and application methods in agriculture. Mechanisms of biostimulation that influence plant performance, physiology, resilience to abiotic stress as well as the plant microbiome are also outlined. Considering current state-of-the-art, perspectives for future research on microalgae-based biostimulants are discussed, ranging from the development of crop-tailored, highly effective products to their application for increasing sustainability in agriculture.

## Microalgae as a Renewable Source of Plant Biostimulants

Global demographic pressure on agricultural production calls for novel and sustainable approaches toward satisfying the ever-growing demand for plant biomass destined for human food, animal feed, and energy production. Conventional agricultural practice has relied overwhelmingly on non-renewable inputs of fertilizers and pesticides. Although their introduction has allowed substantial progress for humankind (Cooper and Dobson, 2007), agro-chemicals also pose a serious, unresolved threat to human health and the environment (Fenner et al., 2013). Moreover, supply and application of these inputs is becoming increasingly costly because of resource depletion and the increasing global demand for mineral fertilizers. Finally, the use of chemical inputs in agriculture is restricted by a tightening legal framework because of mounting public concern. Plant biostimulants (PBs) can play a pivotal role in addressing sustainability challenges because they can reduce dependency on fertilizers, especially on off-farm chemical inputs. Moreover, PBs are also useful to improve yield and its stability under environmental stress (Bulgari et al., 2014; Calvo et al., 2014; Colla and Rouphael, 2015; Yakhin et al., 2017).

According to recent EU Regulation, PBs are defined mainly through their claimed action, in other words ‘*more by the plant response they elicit than by their makeup.*’ Therefore, PBs embrace a wide range of organic non-microbial substances (humic acids, protein hydrolysates, and seaweed extracts) and microbial organisms (mycorrhizal fungi and N-fixing bacteria) (du Jardin, 2015; European Commission, 2016; Rouphael and Colla, 2018; Rouphael et al., 2018). Extracts of brown, green and red macroalgae (seaweed), which are mainly collected from sea water, represent an important category of organic biostimulants (Battacharyya et al., 2015; Rouphael et al., 2017). A wide array of chemicals has been identified in seaweed extracts, including polysaccharides, phenolics, fatty acids, vitamins, osmolytes, phytohormones, and hormone-like compounds involved in signaling plant response to abiotic stress (Khan et al., 2009; Battacharyya et al., 2015). Currently, brown macroalgae are the most common seaweed used in agriculture with dozens of commercial products (Craigie, 2011). The beneficial effects of seaweed extract applications, attributed to multiple mechanisms, include improvement of seedling establishment, flowering and fruit setting, as well as tolerance to a wide range of abiotic stress (Khan et al., 2009; Craigie, 2011; Battacharyya et al., 2015; De Pascale et al., 2018; Ertani et al., 2018). However, macroalgae are typically harvested from sea, which hampers the standardization of the raw material quality. The chemical composition of seaweed varies according to the tissue age, environmental conditions, nutrient availability, and time of harvesting (Marinho-Soriano et al., 2006; Marsham et al., 2007). Moreover, the spread of water pollution limits the use of seaweed biomass for biostimulant production in many parts of the globe. For this reason, the scientific community and the private industry are giving more emphasis to the more costly controlled production of ‘*in-house algae.*’ Ideally, a successful biostimulant should be not only sustainable and effective, but also based on organic by-products and able to favor the closure of the nutrient loops in agriculture.

A promising alternative toward the standardization of raw material and cost reduction in algal biomass production would be the use of microalgae (Barone et al., 2018a,b). They embrace a wide range of evolutionarily diverse phototrophic and unicellular organisms. It has been estimated that the number of microalgae specie ranges from 50,000 to 1 million. Their long and complex evolutionary history is reflected in the vast range of metabolic compounds that they are able to produce. In any aquatic eco-system, microalgae are among the most important primary trophic producers (Sharma and Rai, 2011). They have successfully colonized also terrestrial niches, where they can be found in population densities ranging between 3 and 100 million cells per gram of topsoil (Kutílek and Nielsen, 2015). The very wide genetic variability in these microorganisms has yet to be fully explored and microalgae-based commercial products are currently used mainly as nutritional supplements. Presently, only few microalgal species (*Chlorella* spp., *Dunaliella* spp., *Haematoccocus* spp., *Isochrysis* spp., *Nannochloropsis* spp., *Porphyridium* spp., and *Spirulina* spp.) are industrially exploited (Walker et al., 2005; Wijffels et al., 2013). For instance, *Spirulina* spp. and *Chlorella* spp. have a large economic value for the production of functional food and dietary supplements (Schiavon et al., 2017); while *Dunaliella salina* and *Haematococcus pluvialis* are used to obtain two popular antioxidants, β-carotene and astaxanthin (Bajpai et al., 2013). Microalgae have also gained increasing interest as a source of renewable energy (e.g., biofuel production) and for industrial and domestic wastewater bioremediation (Spolaore et al., 2006; Chiaiese et al., 2011; Renuka et al., 2018).

Microalgae can be autotrophic or heterotrophic. As solar conversion in some microalgae species is very efficient, the most common procedure for cultivation of this microorganism is presently the autotrophic growth (Renuka et al., 2018). The basic cultivation system consists of open-ponds used for food supplement and antioxidant production, with highly variable productivity depending on species and environmental conditions. Open system cultivation of microalgae is thus limited to certain robust species, such as *Spirulina* spp., *Dunaliella* spp., and *Chlorella* spp., that are able to grow under extreme conditions. Reduction of growing area and protection against potential contamination can be obtained in closed-ponds, referred to as photobioreactors. This type of cultivation method is often used for the production of high added-value molecules, such as pharmaceutical compounds. However, the main disadvantage of photobioreactors is the high capital cost for designing and operating. A viable alternative for growing microalgae is in heterotrophic conditions exploiting existing industrial bacterial-bioreactors (Kim et al., 2013; Kovar et al., 2014; Venkata Mohan et al., 2015; Hu et al., 2018). The main advantages of this cultivation system is the high cell concentration, which can reach up to 100 g L^-1^; in photobioreactors, the maximum density is around 40 g L^-1^, even lower in open-ponds (*c*. 10 g L^-1^). A recent sustainable energy-based strategy for growing microalgae relies on using wastewater of industrial, domestic and agricultural origin in bioreactors that allow for the removal of contaminants during the production of microalgal biomass. Therefore, microalgae have the potential to reduce the negative discharges to the environment by, for instance, re-using nutrients and products and valorizing waste from different sources, including those related to agriculture.

This mini-review presents the various methods of production of microalgal extracts, their biologically active compounds and application methods in agriculture. The biostimulant action by which they affect plant performance, physiological status, resilience to environmental stressors as well as their effects on plant microbiome are also covered. Several perspectives for future research relevant to microalgae-based biostimulants are proposed, encompassing the development of specific and effective products as well as their applications for advancing sustainability in modern agriculture.

## Extraction Techniques, Chemical Characterization and Application Methods of Microalgae-Derived Biostimulants

Numerous studies have been carried out on techniques to obtain microalgal extracts (Samarasinghe et al., 2012; Michalak and Chojnacka, 2014, 2015 and refrences cited therein). The extraction techniques require as a first step the removal of the cell wall for releasing the bioactive compounds (Samarasinghe et al., 2012). This can be achieved by: (i) mechanical/physical means (e.g., autoclaving, homogenization, microwaving, pulsed electric field technology, sonication, liquid nitrogen), (ii) chemical means (e.g., sodium hydroxide, hydrochloric or sulfuric acids, osmotic shock, nitrous acid), and (iii) enzymatic means (e.g., cellulase, protease). Mechanical/physical pretreatment methods are more energy-demanding, while enzymatic methods are gaining popularity within the industry, although cell lysing enzymes represent an additional cost (Michalak and Chojnacka, 2014). The significant advantages of the enzymatic pretreatment methods over the chemical ones have been attributed mainly to the more gentle cell-wall disruption, not involving chemical and/or physical treatment. It is therefore expected that the resulting algal extracts will retain higher levels of bioactive compounds (Michalak and Chojnacka, 2014, 2015).

The choice of extraction method used to obtain biologically active compounds from the microalgal biomass is mainly dictated by the type of raw material and by the target molecule(s) (Michalak and Chojnacka, 2014). Conventional techniques include the use of organic solvents. The main drawbacks of traditional solvent extraction techniques are the high volumes of solvents used, the lack of high throughput and the length of the process. Novel extraction methods such as microwave assisted extraction, pressured liquid extraction, supercritical fluid extraction and ultrasound assisted extraction have been adopted, enabling the delivery of extracts in a solvent free-environment, safer for both plants and humans (Michalak and Chojnacka, 2014, 2015).

Microalgae produce a remarkable diversity of biologically active metabolites. The quantity and quality of bioactive metabolites in microalgal extracts largely depend on the extraction technique and the microalgal species used (Puglisi et al., 2018). The content of primary metabolites, carbohydrates and lipids in microalgae is very high (55–70% fresh weight) when they are cultivated under optimal conditions. Carbohydrates are usually one of the major components of microalgal extracts. In *Chlorella* spp., *Chlamydomonas* spp., *Dunaliella* spp. and *Spirulina* spp., carbohydrates may account for up to 46% of the dry weight (DW) extract (Spolaore et al., 2006; Pinzon et al., 2014; Tibbetts et al., 2015). In addition to common carbohydrates, microalgae can contain floridean, myxophycean, and chrysolaminarin starch (Lee, 2008). Protein usually accounts for 18–46% (DW) of microalgal extract (Becker, 2013). The presence of some amino acids such as tryptophan and arginine in microalgal extracts is expected to increase significantly the growth and yield of cultivated crops because these two amino acids are the metabolic precursors of key phytohormones (Colla et al., 2013, 2014, 2016). Tryptophan is pivotal for plant metabolism as it serves as building block for proteins, precursors of plant hormones such as auxin and salicylic acid, and for aromatic secondary compounds with multiple biological functions (Colla et al., 2016). In addition to tryptophan, arginine serves as precursor to polyamines, which partake in many important biological processes such as embryogenesis, organogenesis (particularly flower initiation and development, fruit setting, ripening, and leaf senescence), as well as in protection against osmotic stress (Kalamaki et al., 2009).

To deliver microalgae to agronomic and horticultural crops, a number of application methods have been adopted, depending on the microalgal product and formulation. The modes of application include: (i) soil amendment with algal formulations using suitable carriers, (ii) soil amendment with algal dry biomass (e.g., pellets, granules or powder) or suspended liquid culture, and (iii) foliar spray (using culture supernatant, leachate, and algal compost tea) or substrate/soil drench with algal culture (Coppens et al., 2015; Bushong et al., 2016; Renuka et al., 2018). The foliar application method appeared to be the most effective if applied under high relative humidity conditions and when leaf stomata are open, in order to increase the permeability and uptake of the product.

## Biostimulatory Action of Microalgal Extracts

Microalgae-derived products have multi-functional properties in agriculture, facilitating nutrient uptake, improving crop performance, physiological status and tolerance to abiotic stress (Renuka et al., 2018). Despite the fact that microalgae produce compounds that are active on higher plants (de Morais et al., 2015; Borowitzka, 2016), the practical applications of microalgae in crop science are limited.

In recent years, experimental studies testing the action of microalgal extracts, under open-field and greenhouse conditions, have demonstrated that they stimulate germination, seedling growth, shoot, and root biomass in several crops such as lettuce, red amaranth, pack choi, tomato, and pepper (Faheed and Abd-El Fattah, 2008; Garcia-Gonzalez and Sommerfeld, 2016; Barone et al., 2018a,b; El Arroussi et al., 2018). Promotion of growth (on both fresh and dry weight basis) at the early stages of development was reported for lettuce germinated in a *C. vulgaris* containing medium (at 2 and 3 g dry microalgae extract kg^-1^ of soil) (Faheed and Abd-El Fattah, 2008). In the same study, improvement of plant growth (i.e., shoot, root dry weight, and length) was associated to the stimulation of carotenoid and chlorophyll pigment biosynthesis, which may have improved the photosynthetic activity. Similarly, the application of *Spirulina platensis* enhanced plant growth in different leafy vegetables such as rocket, bayam red, and pak choi (Wuang et al., 2016). Garcia-Gonzalez and Sommerfeld (2016) and El Arroussi et al. (2018) indicated that also fruit vegetables, such as tomato and pepper, are positively affected by the application of microalgal extracts. For instance, seed priming and foliar application at different concentrations (0, 0.75, 1.875, 3.75, 5.625, and 7.5 g mL^-1^) of aqueous cell extracts or dry biomass of green alga *Acutodesmus dimorphus* promoted seed germination, plant growth, and floral production in a dose-dependent manner (Garcia-Gonzalez and Sommerfeld, 2016). Interestingly, the authors demonstrated the presence of a bell-shaped concentration–response curve, with maximum benefit at 3.75 g mL^-1^ extract. Spraying at 3-day intervals with the polysaccharide extract of blue-green alga *S. platensis* increased plant size, root dry weight, the size and number of nodes up to 100%, for tomato and up to 50% for pepper (El Arroussi et al., 2018).

Characterization of the biostimulant action conferred by the extracts of microalgae *C. vulgaris* or *Scenedesmus quadricauda* applied at two concentrations (2 and 4 ml L^-1^) on sugar beet grown in a hydroponic Hoagland solution was carried out by Barone et al. (2018a). Treated seedlings incurred changes in root architecture (higher total root length, surface, and number of root tips). In addition, differences between the two application rates were not observed. The different changes induced by microalgal extracts on root morphology have been reflected also at the molecular level with an upregulation of several genes involved in various biological pathways of primary and secondary metabolism. Zhang et al. (2017) and Barone et al. (2018b) suggested that the hydroponic co-cultivation of tomato plants with *C. vulgaris*, *S. quadricauda* or *C. infusionum* had a positive effect on crop performance in terms of fresh and dry plant weight. A putative biostimulatory mechanism involved may be associated to the continuous algal photosynthesis constantly delivering oxygen to the hydroponic nutrient solution (Barone et al., 2018a). Another possible mechanism involved in the biostimulatory action of microalgae belonging to *Chlorophyta* spp. and *Cyanophyta* spp. is the production and excretion of hormones (auxins and cytokynins) into the growing substrate/soil and surrounding environment (Jäger et al., 2010; Renuka et al., 2018). Microalgal extract applications can also mitigate the detrimental effects imposed on crops by the two major agents of abiotic stress, salinity, and drought (Abd El-Baky et al., 2010; El Arroussi et al., 2018). For instance, El Arroussi et al. (2018) reported that *D. salina* exopolysaccharides mitigate the effect of different salinity levels in tomato by increasing the antioxidant enzymatic activity, phenolic compounds and key metabolites such as neophytadiene, tocopherol, stigmasterol, and 2,4-ditert-butylphenol, which are considered components of the main mechanisms against oxidative stress. The application of aqueous extracts of *Chlorella ellipsoida* and *Spirulina maxima* on wheat (Abd El-Baky et al., 2010) and *Nannochloris* on tomato (Oancea et al., 2013) also mitigated salt stress impact.

Microalgae-based plant biostimulation could be attributed also to the modulation of microbial communities residing in both the phyllosphere and the rhizosphere (Ranjan et al., 2016; Renuka et al., 2018). For instance, inoculation with blue-green algae such as *Calothrix elenkinii* stimulated the phyllosphere and rhizosphere microbiomes (Priya et al., 2015; Manjunath et al., 2016). One of the main mechanisms responsible for the improvement of soil microbial communities in response to inoculation with blue-green algae relates to the production of exopolysaccharides. Exopolysaccharides secreted by many microalgal species provide organic carbon for the growth and development of beneficial microbes, leading to the formation of useful biofilms in the rhizosphere (Xiao and Zheng, 2016). Their association with soil elements helps in the solubilization, mineralization, and bioavailability of macro and micronutrients, thus improving crop performance (Drever and Stillings, 1997).

## Concluding Remarks and Challenges Ahead

The main advantage of microalgae-based biostimulants is that their production requires limited non-renewable resources and bears an overall reduced environmental impact. Compared to other photosynthetic organisms, microalgae are potentially more suitable for biotechnological improvement, especially for metabolic engineering (Fu et al., 2016; Guiheneuf et al., 2016; Jagadevan et al., 2018). However, the advancement of their applications in agriculture is hampered by various factors. While there is a general consensus on the potential benefits of the interaction between microalgae and crops, there is limited scientific evidence underpinning this interaction, compared to other organic/inorganic and microbial PBs. The vast diversity of microscopic algae still remains largely unexplored and little work has been done for the selection and genetic improvement of microalgae accession for agriculture.

In the coming years, research efforts should focus on: (1) elucidating the microalgae × plant species × environment interaction, in order to select optimal combinations; (2) optimizing application parameters (e.g., timing, mode, rate of application, and plant developmental stage); (3) quantitative and qualitative characterization of microbial communities as modulated by microalgal PBs; (4) determining the persistence of effects subsequent to microalgal PBs foliar application; (5) the impact of climatic (e.g., radiation, and relative humidity) and plant morphological factors (e.g., cuticle thickness and leaf permeability) on the effectiveness of microalgal PBs; (6) developing tailored microalgal strains with compositions and formulations adapted to specific environments and (7) identifying potentially synergistic green/blue-green microalgal combinations providing complementary traits (e.g., production of phytohormones, and siderophores, N fixation). Finally, the synergistic effects among green and blue-green algae should be at the centre of future research aiming to design and develop efficient microalgae-based products with specific biostimulation action.

## Author Contributions

All authors listed have made a substantial, direct and intellectual contribution to the work, and approved it for publication.

## Conflict of Interest Statement

The authors declare that the research was conducted in the absence of any commercial or financial relationships that could be construed as a potential conflict of interest.

## References

[B1] Abd El-Baky H. H. Rl-Baz F. K. El Baroty G. S. (2010). Enhancing antioxidant availability in wheat grains from plants grown under seawater stress in response to microalgae extract treatments. *J. Sci. Food Agric.* 90 299–303. 10.1002/jsfa.3815 20355046

[B2] Bajpai R. Prokop A. Zappi M. (2013). *Algal Biorefineries: Volume 1: Cultivation of Cells and Products*. Berlin: Springer, 10.1007/978-94-007-7494-0

[B3] Barone V. Baglieri A. Stevanato P. Broccanello C. Bertoldo G. Bertaggia M. (2018a). Root morphological and molecular responses induced by microalgae extracts in sugar beet (*Beta vulgaris* L.). *J. Appl. Phycol.* 30 1061–1072. 10.1007/s10811-017-1283-3

[B4] Barone V. Puglisi I. Fragalà F. Lo Piero A. R. Giuffrida F. Baglieri A. (2018b). Novel bioprocess for the cultivation of microalgae in hydroponic growing system of tomato plants. *J. Appl. Phycol.* 10.1007/s10811-018-1518-y

[B5] Battacharyya D. Babgohari M. Z. Rathor P. Prithiviraj B. (2015). Seaweed extracts as biostimulants in horticulture. *Sci. Hortic.* 196 39–48. 10.1016/j.scienta.2015.09.012 27826310

[B6] Becker E. W. (2013). “Microalgae in human and animal nutrition,” in *Handbook of Microalgal Culture*, eds Richmond A. Hu Q. (Hoboken, NJ: Wiley), 461–503. 10.1002/9781118567166.ch25

[B7] Borowitzka M. A. (2016). “Chemically-mediated interactions in microalgae,” in *The Physiology of Microalgae*, eds Borowitzka M. A. Beardall J. Raven J. A. (Cham: Springer International Publishing), 321–357.

[B8] Bulgari R. Cocetta G. Trivellini A. Vernieri P. Ferrante A. (2014). Biostimulants and crop responses: a review. *Biol. Agric. Hortic.* 31 1–17. 10.1080/01448765.2014.964649

[B9] Bushong J. T. Miller E. C. Mullock J. L. Arnall D. B. Raun W. R. (2016). Irrigated and rain-fed maize response to different nitrogen fertilizer application methods. *J. Plant Nutr.* 39 1874–1890. 10.1080/01904167.2016.1187747

[B10] Calvo P. Nelson L. Kloepper J. W. (2014). Agricultural uses of plant biostimulants. *Plant Soil* 383 3–41. 10.1007/s11104-014-2131-8

[B11] Chiaiese P. Palomba F. Tatino F. Lanzillo C. Pinto G. Pollio A. (2011). Engineered tobacco and microalgae secreting the fungal laccase POXA1b reduce phenol content in olive oil mill wastewater. *Enzyme Microb. Technol.* 49 540–546. 10.1016/j.enzmictec.2011.06.002 22142729

[B12] Colla G. Rouphael Y. (2015). Biostimulants in horticulture. *Sci. Hortic.* 196 1–2. 10.1016/j.scienta.2015.10.044

[B13] Colla G. Rouphael Y. Canaguier R. Svecova E. Cardarelli M. (2014). Biostimulant action of a plant-derived protein hydrolysate produced through enzymatic hydrolysis. *Front. Plant Sci.* 5:448. 10.3389/fpls.2014.00448 25250039 PMC4158787

[B14] Colla G. Rouphael Y. Lucini L. Canaguier R. Stefanoni W. Fiorillo A. (2016). Protein hydrolysate-based biostimulants: origin, biological activity and application methods. *Acta Hortic.* 1148 27–34. 10.17660/ActaHortic.2016.1148.3

[B15] Colla G. Svecova E. Rouphael Y. Cardarelli M. Reynaud H. Canaguier R. (2013). Effectiveness of a plant-derived protein hydrolysate to improve crop performances under different growing conditions. *Acta Hortic.* 1009 175–179. 10.17660/actahortic.2013.1009.21

[B16] Cooper J. Dobson H. (2007). The benefits of pesticides to mankind and the environment. *Crop Pro.* 26 1337–1348. 10.1016/j.cropro.2007.03.022

[B17] Coppens J. Grunert O. Van Den Hende S. Vanhoutte I. Boon N. Haesaert G. (2015). The use of microalgae as a high-value organic slow-release fertilizer results in tomatoes with increased carotenoid and sugar levels. *J. Appl. Phycol.* 28 2367–2377. 10.1007/s10811-015-0775-2

[B18] Craigie J. S. (2011). Seaweed extract stimuli in plant science and agricolture. *J. Appl. Phycol.* 23 371–393. 10.1007/s10811-010-9560-4

[B19] de Morais M. G. da Silva Vaz B. de Morais E. G. Costa J. A. V. (2015). Biologically active metabolites synthesized by microalgae. *Bio. Med. Res. Inter.* 2015:835761. 10.1155/2015/835761 26339647 PMC4538420

[B20] De Pascale S. Rouphael Y. Colla G. (2018). Plant biostimulants: innovative tool for enhancing plant nutrition in organic farming. *Eur. J. Hortic. Sci.* 82 277–285. 10.17660/eJHS.2017/82.6.2

[B21] Drever J. I. Stillings L. L. (1997). The role of organic acids in mineral weathering. *Colloids Surf. Physicochem. Eng. Asp.* 120 167–181. 10.1016/S0927-7757(96)03720-X

[B22] du Jardin P. (2015). Plant biostimulants: definition, concept, main categories and regulation. *Sci. Hortic.* 196 3–14. 10.1016/j.scienta.2015.09.021

[B23] El Arroussi H. Benhima R. Elbaouchi A. Sijilmassi B. El Mernissi N. Aafsar A. (2018). *Dunaliella salina* exopolysaccharides: a promising biostimulant for salt stress tolerance in tomato (*Solanum lycopersicum*). *J. Appl. Phycol.* 30 2929–2941. 10.1007/s10811-017-1382-1

[B24] Ertani A. Francioso O. Tinti A. Schiavon M. Pizzeghello D. Nardi S. (2018). Evaluation of seaweed extracts from *Laminaria* and *Ascophyllum nodosum* spp. as biostimulants in *Zea mays* L. Using a combination of chemical, biochemical and morphological approaches. *Front. Plant Sci.* 9:428. 10.3389/fpls.2018.00428 29681909 PMC5897654

[B25] European Commission (2016). *Proposal for a Regulation Laying Down Rules on the Making Available on the Market of Ce Marked Fertilising Products and Amending Regulations* Technical Report EC 1069/2009 and EC1107/2009. Brussels: European Commission

[B26] Faheed F. A. Abd-El Fattah Z. (2008). Effect of *Chlorella vulgaris* as bio-fertilizer on growth parameters and metabolomic aspects of lettuce plant. *J. Agric. Soc. Sci.* 4 165–169.

[B27] Fenner K. Canonica S. Wackett L. P. Elsner M. (2013). Evaluating pesticide degradation in the environment: blind spots and emerging opportunities. *Science* 341 752–758. 10.1126/science.1236281 23950532

[B28] Fu W. Chaiboonchoe A. Khraiwesh B. Nelson D. R. Al-Khairy D. Mystikou A. (2016). Algal cell factories: approaches, applications, and potentials. *Mar. Drugs* 14:225. 10.3390/md14120225 27983586 PMC5192462

[B29] Garcia-Gonzalez J. Sommerfeld M. (2016). Biofertilizer and biostimulant properties of the microalgae *Acutodesmus dimorphus*. *J. Appl. Phycol.* 28 1051–1061. 10.1007/s10811-015-0625-2 27057088 PMC4789255

[B30] Guiheneuf F. Khan A. Tran L. S. (2016). Genetic engineering: a promising tool to engender physiological, biochemical, and molecular stress resilience in green microalgae. *Front. Plant Sci.* 31:400. 10.3389/fpls.2016.00400 27066043 PMC4815356

[B31] Hu J. Nagarajan D. Zhang Q. Chang J. S. Lee D. J. (2018). Heterotrophic cultivation of microalgae for pigment production: a review. *Biotechnol. Adv.* 36 54–67. 10.1016/j.biotechadv.2017.09.009 28947090

[B32] Jagadevan S. Banerjee A. Banerjee C. Guria C. Tiwari R. Baweja M. (2018). Recent developments in synthetic biology and metabolic engineering in microalgae towards biofuel production. *Biotechnol. Biofuels* 11:185. 10.1186/s13068-018-1181-1 29988523 PMC6026345

[B33] Jäger K. Bartók T. Ördög V. Barnabás B. (2010). Improvement of maize (*Zea mays* L.) anther culture responses by algae-derived natural substances. *S. Afr. J. Bot.* 76 511–516. 10.1016/j.sajb.2010.03.009

[B34] Kalamaki M. S. Merkouropoulos G. Kanellis A. K. (2009). Can ornithine accumulation modulate abiotic stress tolerance in Arabidopsis? *Plant Signal. Behav.* 4 1099–1101. 10.4161/psb.4.11.9873 19901538 PMC2819526

[B35] Khan W. Rayirath U. P. Subramanian S. Jithesh M. N. Rayorath P. Hodges D. M. (2009). Seaweed extracts as biostimulants of plant growth and development. *J. Plant Growth Regul.* 28 386–399. 10.1007/s00344-009-9103-x

[B36] Kim S. Park J.-E. Cho Y.-B. Hwang S.-J. (2013). Growth rate, organic carbon and nutrient removal rates of *Chlorella sorokiniana* in autotrophic, heterotrophic and mixotrophic conditions. *Biores. Technol.* 144 8–13. 10.1016/j.biortech.2013.06.068 23850820

[B37] Kovar K. Přibyl P. Wyss M. (2014). “Microalgae grown under heterotrophic and mixotrophic conditions,” in *Industrial Scale Suspension Culture of Living Cells*, eds Meyer H. P. Schmidhalter D. R. (Hoboken, NJ: Wiley), 164–185. 10.1002/9783527683321.ch04

[B38] Kutílek M. Nielsen D. R. (2015). *Soil: The Skin of the Planet Earth*. Berlin: Springer, 13–19. 10.1007/978-94-017-9789-4_3

[B39] Lee R. E. (2008). “Basic characteristics of the algae,” in *Phycology*, 4 Edn, ed. Lee R. E. (Cambridge: Cambridge University Press), 3–30. 10.1017/cbo9780511812897.002

[B40] Manjunath M. Kanchan A. Ranjan K. Venkatachalam S. Prasanna R. Ramakrishnan B. (2016). Beneficial cyanobacteria and eubacteria synergistically enhance bioavailability of soil nutrients and yield of okra. *Heliyon* 2:e00066. 10.1016/j.heliyon.2016.e00066 27441245 PMC4945968

[B41] Marinho-Soriano E. Fonseca P. C. Carneiro M. A. A. Moreira W. S. C. (2006). Seasonal variation in the chemical composition of two tropical seaweeds. *Biores. Technol.* 97 2402–2406. 10.1016/j.biortech.2005.10.014 16311028

[B42] Marsham S. Scott G. W. Tobin M. L. (2007). Comparison of nutritive chemistry of a range of temperate seaweeds. *Food Chem.* 100 1331–1336. 10.1016/j.foodchem.2005.11.029

[B43] Michalak I. Chojnacka K. (2014). Algal extracts: Technology and advances. *Eng. Life Sci.* 14 581–591. 10.1002/elsc.201400139 11122535

[B44] Michalak I. Chojnacka K. (2015). Algae as production systems of bioactive compounds. *Eng. Life Sci.* 15 160–176. 10.1002/elsc.201400191

[B45] Oancea L. Velea S. Fãtu V. Mincea C. Ilie L. (2013). Micro-algae based plant biostimulant and its effect on water stressed tomato plants. *J. Plant Prot.* 6 104–114.

[B46] Pinzon A. Y. Gonzalez-Delgado A. D. Kafarov V. (2014). Optimization of microalgae composition for development of a typology of biorefinery based on profitability analysis. *Chem. Eng. Trans.* 37 457–462.

[B47] Priya H. Prasanna R. Ramakrishnan B. Bidyarani N. Babu S. Thapa S. (2015). Influence of cyanobacterial inoculation on the culturable microbiome and growth of rice. *Microbiol. Res.* 171 78–89. 10.1016/j.micres.2014.12.011 25644956

[B48] Puglisi I. Barone V. Sidella S. Coppa M. Broccanello C. Gennari M. (2018). Biostimulant activity of humic-like substances from agro-industrial waste of *Chlorella vulgaris* and *Scenedesmus quadricauda*. *Eur. J. Phycol.* 53 433–442. 10.1080/0.9670262.2018.1458997

[B49] Ranjan K. Priya H. Ramakrishnan B. Prasanna R. Venkatachalam S. Thapa S. (2016). Cyanobacterial inoculation modifies the rhizosphere microbiome of rice planted to a tropical alluvial soil. *Appl. Soil Ecol.* 108 195–203. 10.1016/j.apsoil.2016.08.010

[B50] Renuka N. Guldhe A. Prasanna R. Singh P. Bux F. (2018). Microalgae as multi-functional options in modern agriculture: current trends, prospects and challenges. *Biotechnol. Adv.* 36 1255–1273. 10.1016/j.biotechadv.2018.04.004 29673972

[B51] Rouphael Y. Colla G. (2018). Synergistic biostimulatory action: Designing the next generation of plant biostimulants for sustainable agriculture. *Front. Plant Sci.* 9:1655. 10.3389/fpls.2018.01655 30483300 PMC6243119

[B52] Rouphael Y. De Micco V. Arena C. Raimondi G. Colla G. De Pascale S. (2017). Effect of *Ecklonia maxima* seaweed extract on yield, mineral composition, gas exchange, and leaf anatomy of zucchini squash grown under saline conditions. *J. Appl. Phycol.* 29 459–470. 10.1007/s10811-016-0937-x

[B53] Rouphael Y. Spíchal L. Panzarová K. Casa R. Colla G. (2018). High-throughput plant phenotyping for developing novel biostimulants: from lab to field or from field to lab? *Front. Plant Sci.* 9:1197. 10.3389/fpls.2018.01197 30154818 PMC6102389

[B54] Samarasinghe N. Fernando S. Faulkner B. (2012). Effect of high pressure homogenization on aqueous phase solvent extraction of lipids from nannochloris *Oculata microalgae*. *J. Ener. Nat. Res.* 1 1–7. 10.11648/j.jenr.20120101.11

[B55] Schiavon M. Ertani A. Parrasia S. Vecchia F. D. (2017). Selenium accumulation and metabolism in algae. *Aquat. Toxicol.* 189 1–8. 10.1016/j.aquatox.2017.05.011 28554051

[B56] Sharma N. K. Rai A. K. (2011). Biodiversity and biogeography of microalgae: progress and pitfalls. *Environ. Rev.* 19 1–15. 10.1139/a10-020

[B57] Spolaore P. Joannis-Cassan C. Duran E. Ismbert A. (2006). Commercial applications of microalgae. *J. Biosc. Bioneng.* 101 87–96. 10.1263/jbb.101.87 16569602

[B58] Tibbetts S. M. Milley J. E. Lall S. P. (2015). Chemical composition and nutritional properties of fresh water and marine microalgal biomass cultured in photobioreactors. *J. Appl. Phycol.* 27 1109–1119. 10.1007/s10811-014-0428-x

[B59] Venkata Mohan S. Rohit M. V. Chiranjeevi P. Chandra R. Navaneeth B. (2015). Heterotrophic microalgae cultivation to synergize biodiesel production with waste remediation: progress and perspectives. *Biores. Technol.* 184 169–178. 10.1016/j.biortech.2014.10.056 25497058

[B60] Walker T. L. Purton S. Becker D. K. Collet C. (2005). Microalgae as bioreactors. *Plant Cell Rep.* 24 629–641. 10.1007/s00299-005-0004-6 16136314

[B61] Wijffels R. H. Kruse O. Hellingwerf K. J. (2013). Potential of industrial biotechnology with cyanobacteria and eukaryotic microalgae. *Curr. Opin. Biotechnol.* 24 405–413. 10.1016/j.copbio.2013.04.004 23647970

[B62] Wuang S. C. Khin M. C. Chua P. Q. D. Luo Y. D. (2016). Use of Spirulina biomass produced from treatment of aquaculture wastewater as agricultural fertilizers. *Algal Res.* 15 59–64. 10.1016/j.algal.2016.02.009

[B63] Xiao R. Zheng Y. (2016). Overview of microalgal extracellular polymeric substances (EPS) and their applications. *Biotechnol. Adv.* 34 1225–1244. 10.1016/j.biotechadv.2016.08.004 27576096

[B64] Yakhin O. I. Lubyanov A. A. Yakhin I. A. Brown P. H. (2017). Biostimulants in plant science: a global perspective. *Front. Plant Sci.* 7:2049. 10.3389/fpls.2016.02049 28184225 PMC5266735

[B65] Zhang J. Wang X. Zhou Q. (2017). Co-cultivation of *Chlorella spp* and tomato in a hydroponic system. *Biomass. Bioenergy* 97 132–138. 10.1016/j.biombioe.2016.12.024

